# Efficient mining gapped sequential patterns for motifs in biological sequences

**DOI:** 10.1186/1752-0509-7-S4-S7

**Published:** 2013-10-23

**Authors:** Vance Chiang-Chi Liao, Ming-Syan Chen

**Affiliations:** 1 Department of Electrical Engineering, National Taiwan University, Taipei, 10617, Taiwan; 2 Research Center for Information Technology Innovation (CITI), Academia Sinica, Taipei, 11529, Taiwan

## Abstract

**Background:**

Pattern mining for biological sequences is an important problem in bioinformatics and computational biology. Biological data mining yield impact in diverse biological fields, such as discovery of co-occurring biosequences, which is important for biological data analyses. The approaches of mining sequential patterns can discover all-length motifs of biological sequences. Nevertheless, traditional approaches of mining sequential patterns inefficiently mine DNA and protein data since the data have fewer letters and lengthy sequences. Furthermore, gap constraints are important in computational biology since they cope with irrelative regions, which are not conserved in evolution of biological sequences.

**Results:**

We devise an approach to efficiently mine sequential patterns (motifs) with gap constraints in biological sequences. The approach is the *Depth-First Spelling *algorithm for mining sequential patterns of biological sequences with *Gap *constraints (termed *DFSG*).

**Conclusions:**

*PrefixSpan *is one of the most efficient methods in traditional approaches of mining sequential patterns, and it is the basis of *GenPrefixSpan. GenPrefixSpan *is an approach built on *PrefixSpan *with gap constraints, and therefore we compare *DFSG *with *GenPrefixSpan*. In the experimental results, *DFSG *mines biological sequences much faster than *GenPrefixSpan*.

## Background

Pattern mining has numerous applications, such as purchasing pattern mining, biological pattern mining, and Web log pattern mining. Therefore the academic community has devised useful methods to mine patterns, e.g., mining traditional sequential patterns [1][2][3][4], maximal sequential patterns [5], closed sequential patterns [6], sequential patterns of data streams [7], incremental sequential patterns [8], and progressive sequential patterns [9]. Traditional sequential pattern mining methods discover general sequential patterns, which can be applied to various constraints. The methods of mining traditional sequential patterns have two famous types of algorithms from technical view. The two types are apriori-based methods [1][2] and projection-based pattern growth algorithms [3][4]. The apriori-based methods combine items into candidate patterns, and then the methods validate the patterns. The projection-based pattern growth algorithms scan all sequences and project patterns recursively. The data formats of traditional methods are divided into horizontal data formats and vertical data formats.

Traditional sequential pattern mining methods discover 2*^l ^*subsequences of a sequential pattern with length *l*. The numbers of subsequences for a sequential pattern are too large in traditional mining methods, and therefore the maximal sequential pattern mining method [5] is proposed to efficiently identify maximal sequential patterns, which have no frequent supersequences. Another alternative is to mine closed sequential patterns [6], which patterns do not have any frequent supersequences with the same occurrence frequency. The closed sequential patterns not only largely reduce the number of reported sequential patterns, but also preserve the expressive power of traditional mining algorithms since the subsequences of a closed sequential pattern are easily derived. Mining sequential patterns of data streams [7] is in a different environment and has some additional constraints, such as strictly restricted memory, continuously identified sequential patterns, and a linear time execution.

Incremental databases are formed with newly added sequences. The incremental sequential pattern mining algorithm [8] is devised to efficiently mine incremental databases since many real data grow incrementally. Most users are usually interested in recent data, and therefore the progressive sequential pattern mining algorithm [9] generates sequential patterns in a period of interest. The method can find newly arriving sequential patterns and discard obsolete sequential patterns. Data mining technology has been used in bioinformatics domain. For example, temporal pattern mining techniques are used to mine predictive and non-spurious patterns [10]. Associated functional subgraphs are discovered by a pattern mining method [11] in cancer protein-protein interaction networks. It is increasingly important to develop approaches for efficient biological data mining since biological sequences are now in widespread use in the field of bioinformatics.

Some important research directions for data mining in bioinformatics are discovery of co-occurring biological sequences, effectively classifying biological sequences, and clustering biological sequences [12][13][14]. In molecular biology, the motifs are functional significance and have specific structures which are mined from unaligned biological sequences. Mining sequential patterns (motifs) promote identifying co-occurring biological sequences and discovering relationships in DNA or protein data [15][16]. In bioinformatics domain, mining sequential patterns (motifs) have shown the usefulness, such as classification of biological sequences, prediction of transcription factor binding sites, recognition of protein folds, and identification of hot regions in protein-protein interactions.

The problems of mining sequential patterns correlate closely with some traditional problems of computational biology [17][18][19][20], such as the problems of motif finding and those of sequence alignment. In the field of biology, biological sequences conserve sequential patterns for long evolution, which may be critical functions. The *2PDF *approach [21] first proposed to mine sequential patterns of biological sequences, but a large number of patterns were generated, and gap constraints were not coped. *DFSP *[22] is a general model of mining sequential patterns for biological sequences, but it did not cope with gap constraints either. The gap constraints of *TEIRESIAS *algorithm [23] and *SPLASH *[24] are rigid. The *TEIRESIAS *algorithm has two phases. The first phase is the scanning, and the second phase is the convolution. In the scanning phase, *TEIRESIAS *generates all <*L, W*> patterns, which are at least k support. *L *is the number of least residues, and *W *is the length of patterns. In the convolution phase, *TEIRESIAS *constructs maximal patterns from <*L, W*> patterns. *SPLASH *is another algorithm with the rigid gap constraints. *SPLASH *first builds a seed set, and then extends patterns recursively. All the final patterns satisfy the density constraint. The density constraint denotes that all substrings of a pattern have length *l_0 _*and at least *k_0 _*full characters.

Next, we briefly introduce the *2PDF *method. New and different types of patterns are generated by the *2PDF *method. The patterns have the form "P_1_*P_2_*...*P_k_*...*P_n-1_*P_n_." Each "P_i_" denotes a frequent segment, in contrast to the complete set of patterns in traditional sequential pattern mining problems. A frequent segment represents a segment that is longer than *MinLen *(minimum segment length). The arbitrary lengths of items or gaps are represented by one symbol "*". They extract segments from all sequences by a generalized suffix tree. To generate the pattern tree in the *2PDF *method, the segment tree (composed of the segments) is used. The method mines the complete set of sequential patterns in only setting *MinLen *= 1 for the *2PDF *method. The complete set of all-length sequential patterns means the complete set of sequential patterns. The complete set of length 1 sequential patterns in DNA sequences may be {<A>, <T>, <C>, <G>}. When *MinLen *= 1, the segment tree in the *2PDF *method is too large. A combinatorial method generated the pattern tree in the method. Thus, too many patterns (all combinations of the "*" position) are generated by these techniques. For example, the *2PDF *method may generate the patterns "abc*d," "ab*cd," "a*bcd," "ab*c*d," "a*bc*d," "a*b*cd," and "a*b*c*d" if the *DFSG *[25] or *GenPrefixSpan *[26] merely generates the pattern "a*b*c*d" (without limitation of gap constraints). The *2PDF *method mines too many patterns for biological sequences, which are shown in this example.

The traditional algorithms of mining sequential patterns [1][2][3][4] cope with a large number of items and short sequence lengths. Nevertheless, two diverse characteristics are in DNA and protein data. First, the alphabet of DNA data are made up of four letters, and that of protein data are made up of twenty letters. Second, the DNA and protein data usually have hundreds or thousands of the sequence lengths. Accordingly, traditional approaches of mining sequential patterns difficultly cope with small alphabets and lengthy sequences of biological sequences. Consequently, traditional algorithms are ineffective for mining biological sequences. Projection-based pattern growth algorithms [3][4] are used to process long sequences in traditional sequential pattern mining, but they require an extensive running time because they need to construct and scan corresponding projected databases numerous times to generate long sequential patterns. Another type of algorithm, apriori-based methods [1][2], are frequently used in traditional sequential pattern mining, but they also have a long processing time. Moreover, traditional approaches suit to a larger number of items and brief sequences, such as supermarket transactions; accordingly, the traditional approaches inefficiently cope with biological data.

A novel method, the *Depth-First Spelling *algorithm for mining sequential patterns (motifs) with *Gap *constraints in biological sequences (termed *DFSG *[25]), is devised in this work. This paper is mainly added explanations of gap constraints, explanations of various sequential pattern mining approaches, related works for biological sequences, explanations of projection-based pattern growth algorithms, explanations of the counting matrix techniques for the gap constraints, explanations of GenPrefixSpan, explanations of how to gain our real data, more summaries for this work, and references of related works for biological sequences and rewritten from the proceedings version of our article. Gap constraints are contained in *DFSG*, which is a generalization approach. The distance limitation between two separate letters of a sequence is a gap constraint. The gap constraint suits to the data features with fewer letters and lengthy sequences, such as biological sequences. The unrelated sections of the biological evolution are skipped by the gap constraints. For the gap constraints, a maximal number of the distance limitation in the separate letters can be assigned by the user. We devise the *DFSG *approach to leave traditional methods of mining sequential patterns, and struggles with the problems of the long runtime. *DFSG *need briefer runtime to execute to discover motifs of biological data, compared to traditional approaches of mining sequential patterns. The *DFSG *approach was evaluated by a large number of experiments. First, *DFSG *and *GenPrefixSpan *[26] were utilized to cope with real and simulated DNA data. Afterward the executing time of the two approaches was contrasted by using increased values of gap constraints, synthetic protein data, and diversified variables in synthetic biological data. In the experimental results, the runtime of the *DFSG *approach is superior to that of *GenPrefixSpan *in biological data, and *DFSG *is more scalable.

**Figure 1:**
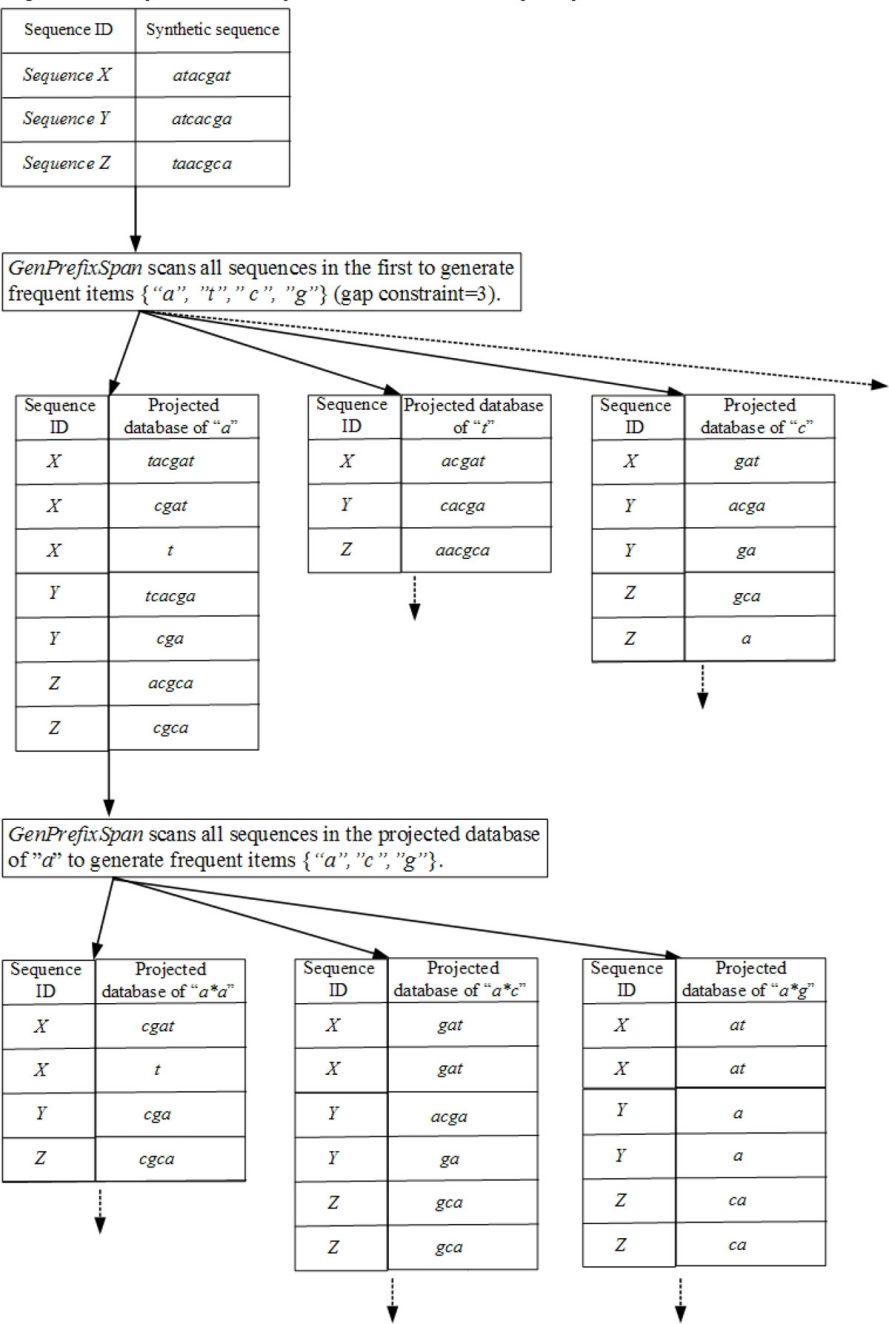
**A partial example of the *GenPrefixSpan *process**.

Some reasons are accounted for the efficient runtime and scalability of the *DFSG *method. We compare the *DFSG *approach with *GenPrefixSpan*, which is a projection-based method of the pattern growth. Corresponding projected databases are not needed to build by the *DFSG *approach unlike traditional projection-based methods of pattern growth; accordingly, the databases are not needed to scan by *DFSG*. Then, *DFSG *saves recursive runtime of projection and scan. As shown in Figure 1, the executing steps of *GenPrefixSpan *are partially exhibited in an example. *GenPrefixSpan *must first scan all sequences to generate frequent items {"a","t","c","g"} in biological sequences {*X: atacgat, Y: atcacga, Z: taacgca*} with the minimum support *λ *equal to *3 *and the gap constraint equal to *3 *(Figure 1). Then, the projected databases for "a", "t", "c", and "g" are generated individually, as {tacgat, cgat, t, tcacga, cga, acgca, cgca}, {acgat, cacga, aacgca}, {gat, acga, ga, gca, a}, and {at, a, ca}, respectively. All the sequences in the projected database of "a" are scanned by *GenPrefixSpan *to generate frequent items {"a","c","g"} after projecting projected database for "a", and then projected databases for "aa," "ac," and "ag" are generated individually. The *GenPrefixSpan *approach projects the corresponding databases recursively until it can not generate any frequent letters.

## Methods

### DFSG algorithm

This section introduces the Depth-First Spelling algorithm for Gapped sequential pattern mining of biological sequences (referred to as *DFSG*). *DFSG *is designed for efficient mining sequential patterns of biological sequences with gap constraints. The gap constraints are critical and have numerous applications in bioinformatics. A counting matrix *C_l _*is proposed to cope with gap constraints and it records each position of a latest item for a gapped sequential pattern in each sequence. The latest item positions in the counting matrix *C_l _*must satisfy the gap constraint. Each position of the latest item for the gapped sequential pattern is recorded in *C_l _*since each position of the latest item may extend to a next sequential pattern with the gap constraint. If the counting matrix records only one position of the latest item in each sequence, the other positions of the latest item may miss chances to contribute support counts for the next sequential pattern with the gap constraint. If the situation causes the support counts of the next gapped sequential pattern to be less than the minimum support counts, the next gapped sequential pattern will not to be generated, and the subsequent gapped sequential patterns will not be discovered either.

**Figure 2:**
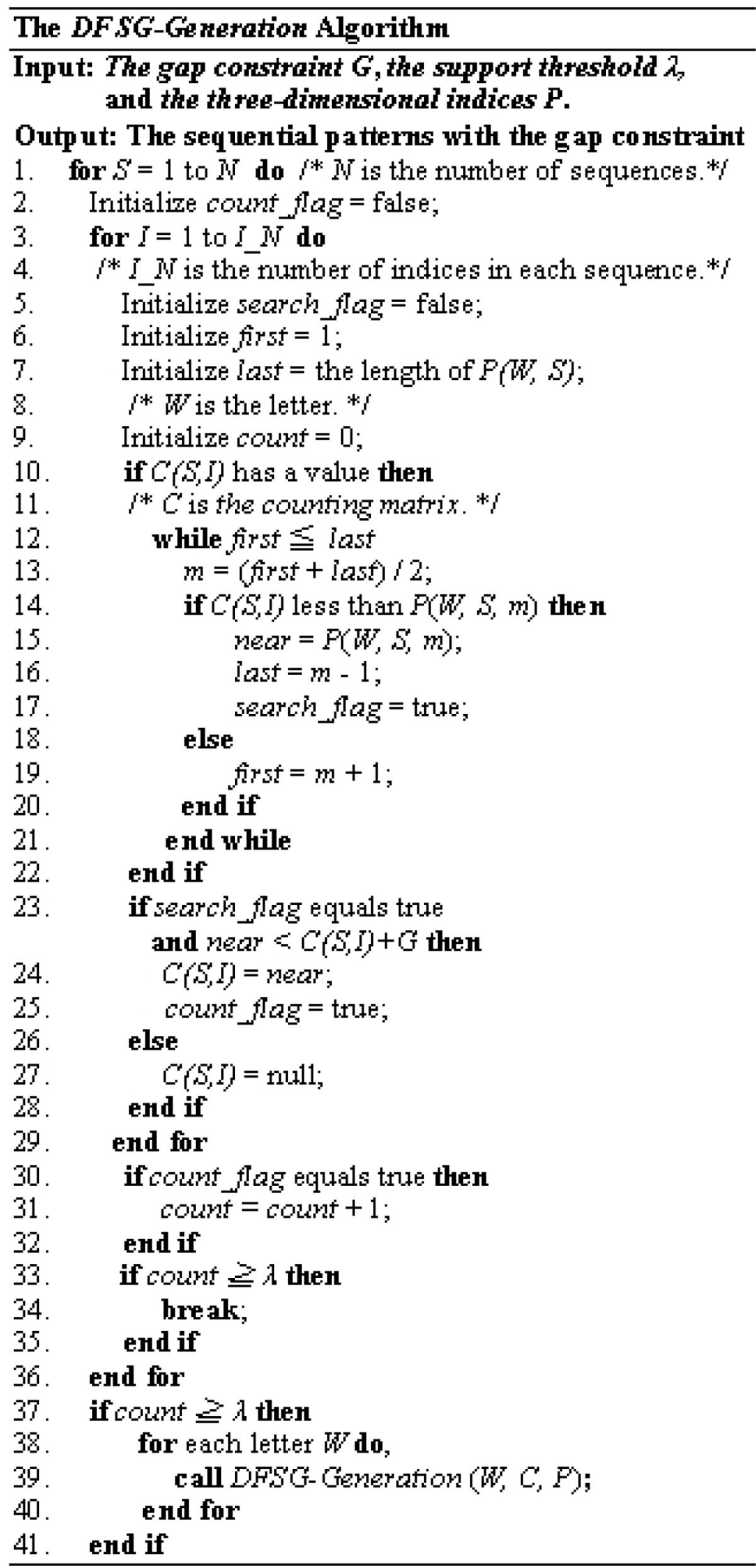
**The *DFSG-Generation *Algorithm**.

All positions of the latest item for a sequence in *C_l _*can contribute only one support count to the support counts of the next gapped sequential pattern since a sequence can contribute only one support count to a pattern. If each position of the latest item for a sequence in *C_l _*can contribute one support count to the next gapped sequential pattern, the support counts of the pattern will be larger, and this situation may result in wrong reported patterns. A sequence can not contribute multiple support counts to a sequential pattern by the definition. In the following, we introduce the execution of the *DFSG *approach. *DFSG *has two performed procedures. First, the three-dimensional indices are built by scanning the provided data set once for the *DFSG *approach. Second, *DFSG-Generation *produces gapped sequential patterns for motifs, as shown in Figure 2. The spelling manner of candidate-gapped patterns and the verification of gapped sequential patterns are included in the *DFSG-Generation *operation. Direct access and binary search with the three-dimensional indices are contained in the procedure of verification. The prefix of each item is depended by the succeeding appearance point of the item for each motif-producing procedure. Therefore, we designed the counting matrix and the three-dimensional indices for the *DFSG-Generation *operation. The counting matrix cannot store the succeeding appearance point ahead since there are too large and unknown feasible points for the succeeding appearance.

**Definition 1**. The set of all items is *E*, which equals {*e_1_, e_2_, ..., e_A_*}. It simulates DNA sequence when *A *equals four. Furthermore, it simulates protein sequence when *A *equals twenty. Let a *sequence ****s ***be the ordered list of items. We denote ***s ***= *{s_1_s_2_s_3_...s_n_}*, where *s_i _*is an item. Biological sequences usually have long lengths, and an identical item can occur many times in a sequence.

**Definition 2**. We denoted a sequence *u *= *{u_1_u_2_u_3_...u_q_}*, where *u_i _*is an item. A sequence *s *contains *u *if *{u_1_u_2_u_3_...u_q_} *is sequentially mapped to *{s_1_s_2_s_3_...s_n_} *(*q≦n*). One *subsequence *of *s *is *u *in the above condition.

**Definition 3**. The *support count *of a pattern *α *is the number of sequences, which contain the pattern *α *in the database. If the support of pattern *α *is larger than the minimum support, this pattern is called a gapped motif. In general, the problem of mining gapped motifs does not confine any categories of biological sequences.

**Definition 4**. We denote a motif *p *= *{p_1_p_2_p_3_...p_m_}*, where *p_m _*is an item. If a sequence *s *can contribute the support to the motif *p*, this motif is one subsequence of the sequence *s*. The item *s_i _*of the sequence is mapped by *p_j_*, and the item *s_k _*of the sequence is mapped by *p_j+1_*. If the *s_k _*position is less than the *s_i _*position plus the gap constraint value, the motif *p *conforms to *the gap constraint G*.

**Definition 5**. *The three-dimensional indices *are the position number *W_k_*, the sequence number *E_j_*, and the item number *A_i_*. The item number *A_i_*, which appears in the sequence number *E_j _*of the biological database is the position number *W_k_*.

**Definition 6**. *The counting matrix C_l _*has multiple position numbers in each sequence number *E_j _*for the procedure of generating gapped motifs. Let *α=<t_1_t_2_...t_n-1_>*be a gapped motif with the suffix *β *in the database that *β=<t_1_t_2_...t_n-1_t_n_>*is a sequence with the prefix *α*. The multiple position numbers form the counting matrix *C_l _*of the suffix *β*. The position number *W_k _*is determined by the item *<t_n_>*in each sequence number *E_j _*of the three-dimensional indices. The position number *W_k _*of the suffix *β *must be greater than the counting matrix *C_l _*of the prefix *α*. The updated position numbers of the present letter in sequences conform to the gap constraint *G *for the gapped motif, and they are recorded by the counting matrix *C_l_*.

**Definition 7**. The *support *of the suffix *β *is the number of rows, which have at least one value in the *counting matrix C_l _*of *β*. If the minimum support is less than the support *γ *of *β*, the sequence *β *is certainly a gapped motif.

### An example of DFSG

**Figure 3:**
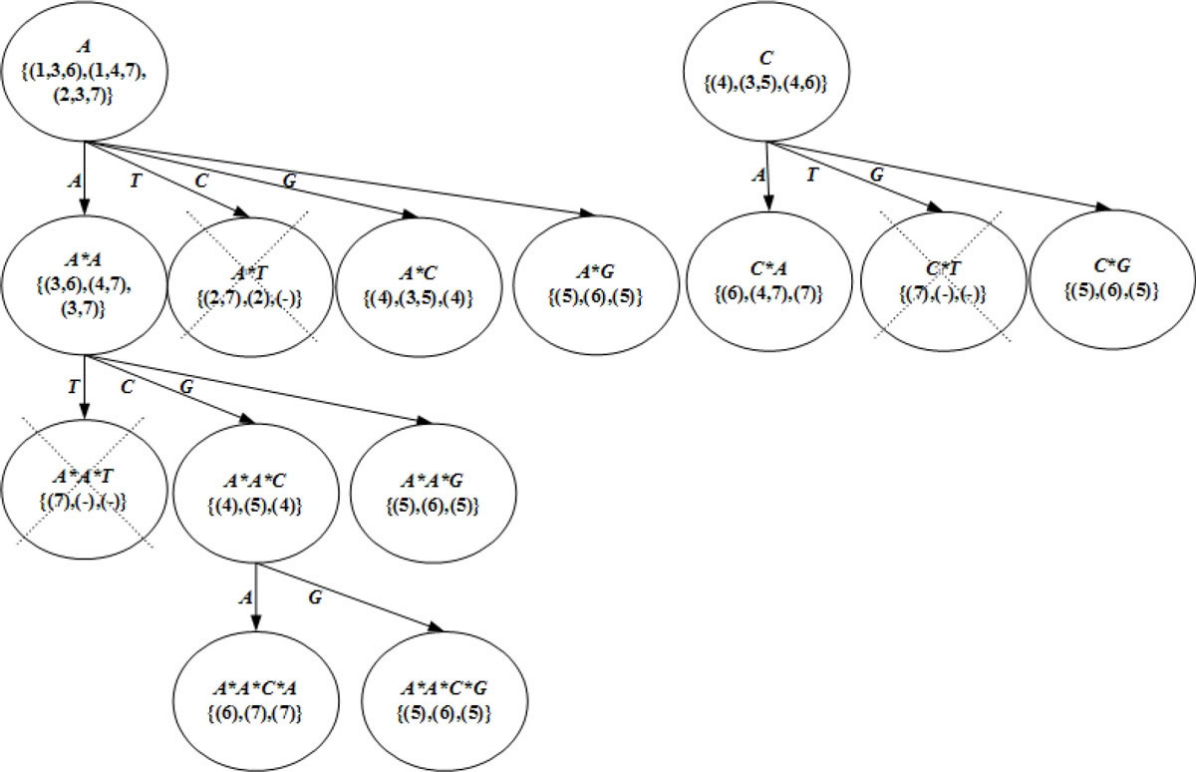
**An example of partial *DFSG-Generation *with *the counting matrix***.

The following is a demonstrated example of performing the *DFSG *approach. A set of items {*A, T, C, G*} for the biological sequence database *D *{*X: ATACGAT, Y: ATCACGA, Z: TAACGCA*} is mined by using the *DFSG *algorithm. For the *DFSG *example, the minimum support *λ *is equal to *3*, and the gap constraint is also equal to *3*. The performed procedures are in the following context. The three-dimensional indices are constructed by using the *DFSG *approach, which reads the biological sequence database once in the first procedure. The *DFSG *approach reads the biological sequence *S*, and puts the position *W_k _*of the read item *A_i _*into the three-dimensional indices. According to Figure 3, the *DFSG-Generation *operation discovers gapped motifs of biological sequences in the second procedure. The *DFSG *approach spells item *I *to generate candidate-gapped motifs α for biological sequences in a depth-first manner. The support counts of candidate-gapped motifs are verified by using the counting matrix *C_l _*and the three-dimensional indices. If the support counts of candidate-gapped motifs are greater than the minimum support count, the recursive execution of the procedure is continued, and the motifs are gapped motifs of biological sequences.

The counting matrix *C_l _*of "*C*" is {(4),(3,5),(4,6)} since "*C*" occurs in the position (4) of sequence *X*, the positions (3,5) of sequence *Y*, and the positions (4,6) of sequence *Z*. In the initial stage, all the positions of "*C*" are in the counting matrix *C_l _*of "*C*" and satisfy the gap constraint. As shown in Figure 3, the candidate-gapped motif "C*A" is spelt by the *DFSG *approach in a depth-first manner. *DFSG *searches the positions in dimension "*A*" of the three-dimensional indices to detect minimum positions that are greater than the positions in the counting matrix and satisfy the gap constraint. The current counting matrix {(6),(4,7),(7)} is greater than the former counting matrix {(4),(3,5),(4,6)}, and all the positions in the new *Cl *satisfy the gap constraint *3 *since the position (6) is less than (9), the positions (4,7) is less than (8,10), and the position (7) is less than (9).

The candidate-gapped motif "C*A" is certainly a gapped motif since the *support count 3 *satisfies the *minimum support count*. A *support *is regarded to satisfy a *minimum support *when the *support *is greater than or equal to the *minimum support*. The *support *of gapped pattern "C*A" is *3 *since the position (6) of sequence *X*, the positions (4,7) of sequence *Y*, and the position (7) of sequence *Z *contribute one support count to the gapped pattern individually. *DFSG *continues to depth-first spell and verify candidate motifs. Then, we observe another candidate motif "A*T." The positions in the "*T*" dimension of the indices are searched. The *support count *is 2 since the updated counting matrix is {(2,7),(2),(-)}. Therefore, the candidate-gapped motif "A*T" is certainly not a gapped motif, and the subsequent candidate-gapped motifs of this failed candidate "A*T" are not continued to generate.

## Results and discussion

### Design of experiments

The *DFSG *performance was evaluated with a number of experiments. In the first part of the experiments, we compared the performance of *DFSG *with that of *GenPrefixSpan *in synthetic and real DNA data. *GenPrefixSpan *is a generalized method of *PrefixSpan*, which uses the projected database approach to recursively construct sequential patterns and is an efficient algorithm in traditional sequential pattern mining. *GenPrefixSpan *stores all subsequences of each frequent item occurrence in projected databases to cope with gap constraints. We acquired real DNA data from the National Center for Biotechnology Information (NCBI), which is national resource funded by U.S. government. In the second part of the experiments, we tested *DFSG *and *GenPrefixSpan *with gap constraints, number of sequences, length of sequences, and simulated protein sequences. The scalability of the *DFSG *algorithm was experimented, too. The total experiments were conducted on a 3.20 GHz Pentium(R) 4 PC with 1 GB of RAM, and Microsoft Windows XP Professional (2002) was the operating system. In order to make fair comparisons, the two programs were written in the same environment, Microsoft Visual C++ 6.0.

### Synthetic and real DNA data

*DFSG *and *GenPrefixSpan *were evaluated by using real DNA data, which are acquired from NCBI. Variables used in the experiments are the length of a sequence *L*, the number of letters *A*, the value of gap constraint *G*, the minimum support *S*, and the number of sequences *N*. The users can access numerous public databases of molecular biology from NCBI website. For example, we introduce how to gain our real data (*A *= 4, *L *= 35, and *N *= 1000). First, we access NCBI website, http://www.ncbi.nlm.nih.gov. Second, the nucleotide database is selected. Third, the query is "sequence AND 35:35[Sequence Length]". Fourth, the first one thousand sequences are crawled and parsed to form our data set.

**Figure 4:**
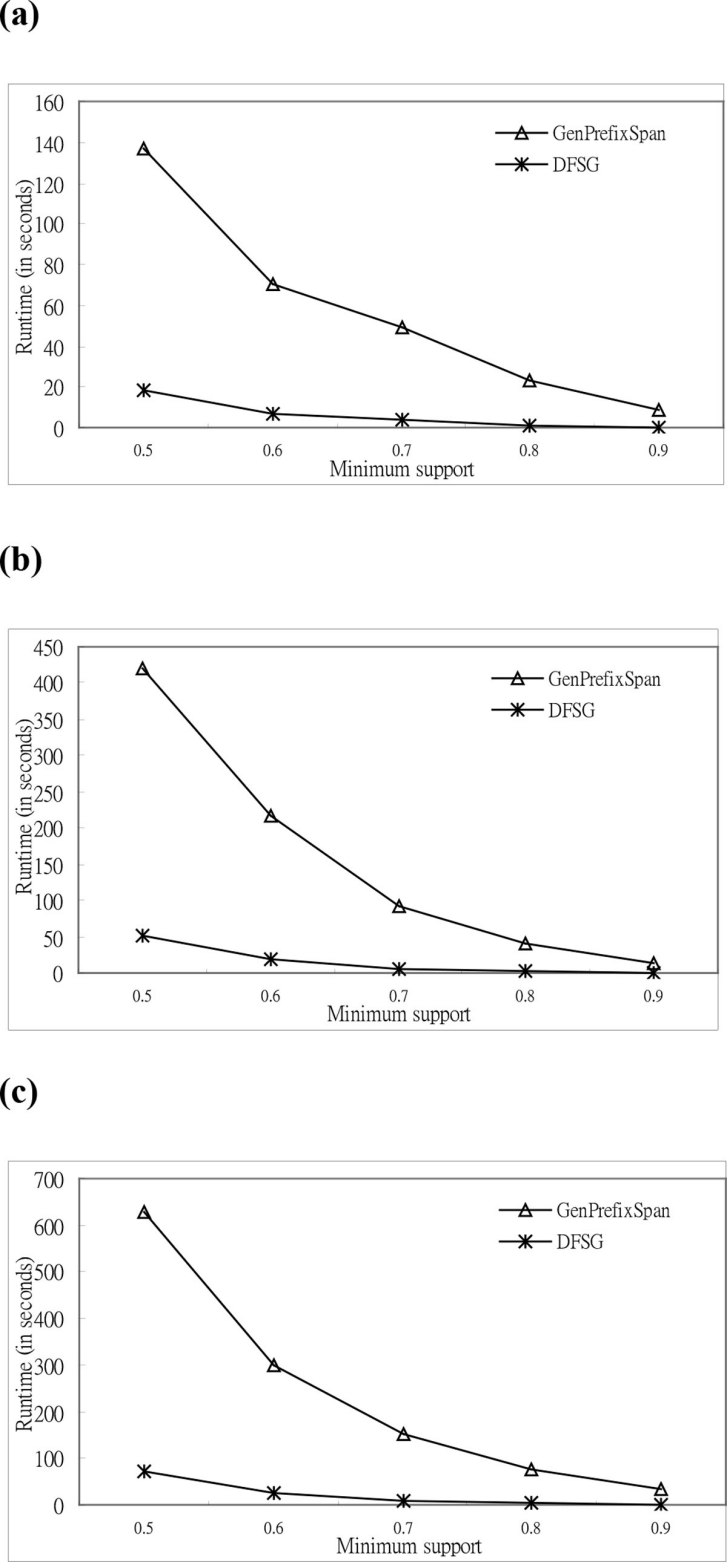
**Comparison of execution time based on real DNA sequences**. **a **Execution Time (*L *= 25, *A *= 4, *G *= 10, and *N=*1000). **b **Execution Time (*L *= 30, *A *= 4, *G *= 7, and *N=*1000). **c **Execution Time (*L *= 35, *A *= 4, *G *= 5, and *N=*1000).

The value of *A *is four for synthetic and real DNA data in the experiments of DNA data. Additionally, the values of *L *are twenty-five, thirty, and thirty-five in the experiments. In Figures 4a-c, *DFSG *is superior to *GenPrefixSpan *for real DNA data. In the experiments, the values of gap constraint are ten, seven, and five; and the number of sequences is one thousand. In the figures, the runtime of two algorithms is shown on the vertical axis, and the minimum support is shown on the horizontal axis. The runtime rate is that the runtime of *GenPrefixSpan *divided by *DFSG*'s runtime. The runtime rates are 8.68, 11.27, 14.77, 20.94, and 30.18 for real DNA data, as shown in Figure 4c. The rate grows when the minimum support gets larger. This means that *DFSG *has more superior than *GenPrefixSpan *for high support thresholds in mining biological sequences.

**Figure 5:**
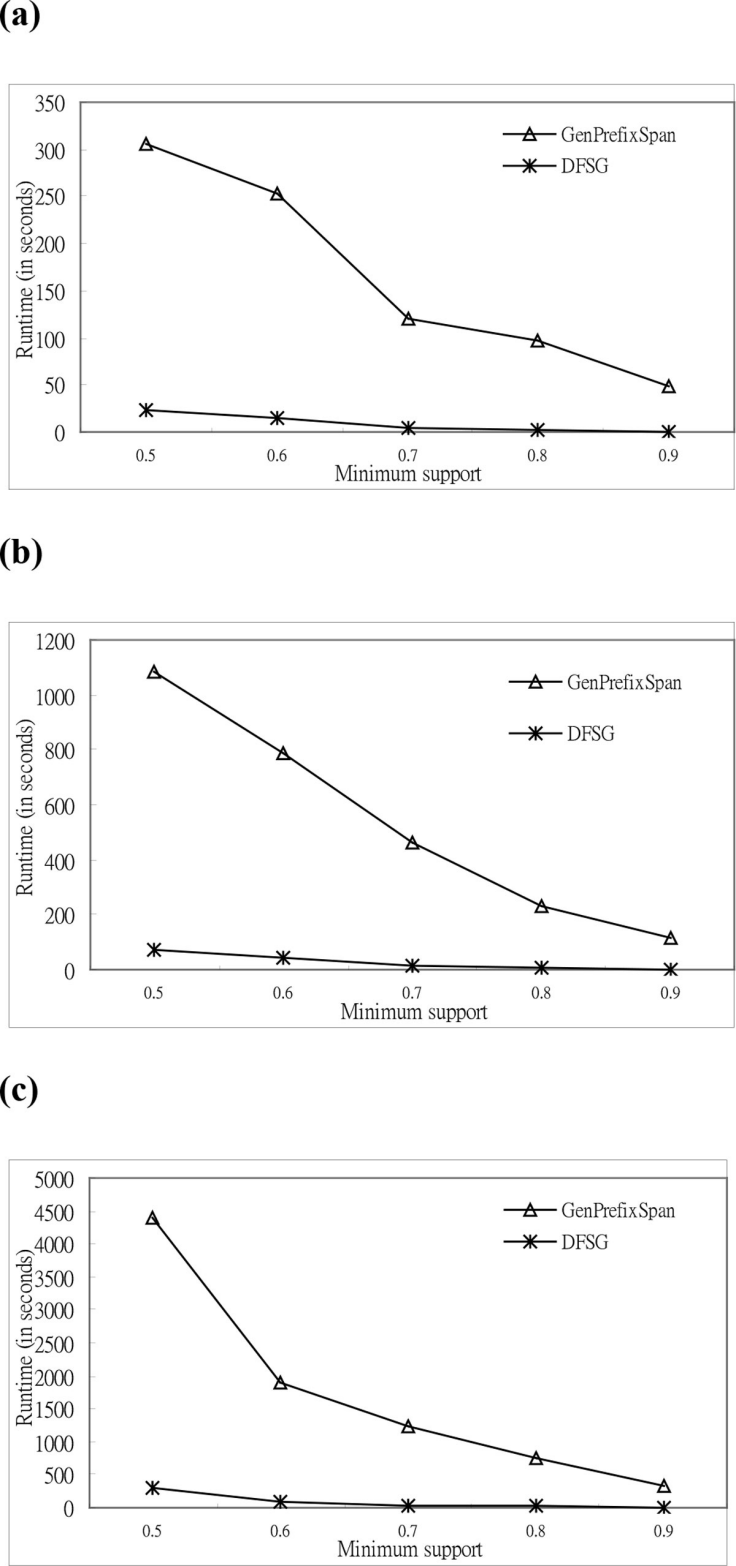
**Comparison of the execution time based on synthetic DNA sequences**. **a **Execution Time (*L *= 25, *A *= 4, *G *= 5, and *N=*3000). **b **Execution Time (*L *= 30, *A *= 4, *G *= 5, and *N=*3000). **c **Execution Time (*L *= 35, *A *= 4, *G *= 5, and *N=*3000).

The simulated DNA data, which is used in the successive experiments, followed the reference [1]. The experimental results of the synthetic DNA data show that *DFSG *is superior to *GenPrefixSpan*, as shown in Figures 5a-c. We simulated the letters for DNA data in the experiments. The number of letters is four; the lengths of the sequences are twenty-five, thirty, and thirty-five; the value of the gap constraint is five; and the number of sequences is three thousand. According to Figure 5c, the runtime rates are 14.04, 22.88, 28.31, 41.41, and 68.93 for synthetic DNA data. The rate grows invariably when the minimum support becomes larger. The performance of *DFSG *for the real DNA data is the same as these for simulated DNA data in the above experiments. This situation confirms that *DFSG *preserves efficiency on real biological data, and simulated sequences can validate the performance of *DFSG *correctly.

### Gap constraints, simulated protein sequences, number of sequences, length of sequences, and scalability

**Figure 6:**
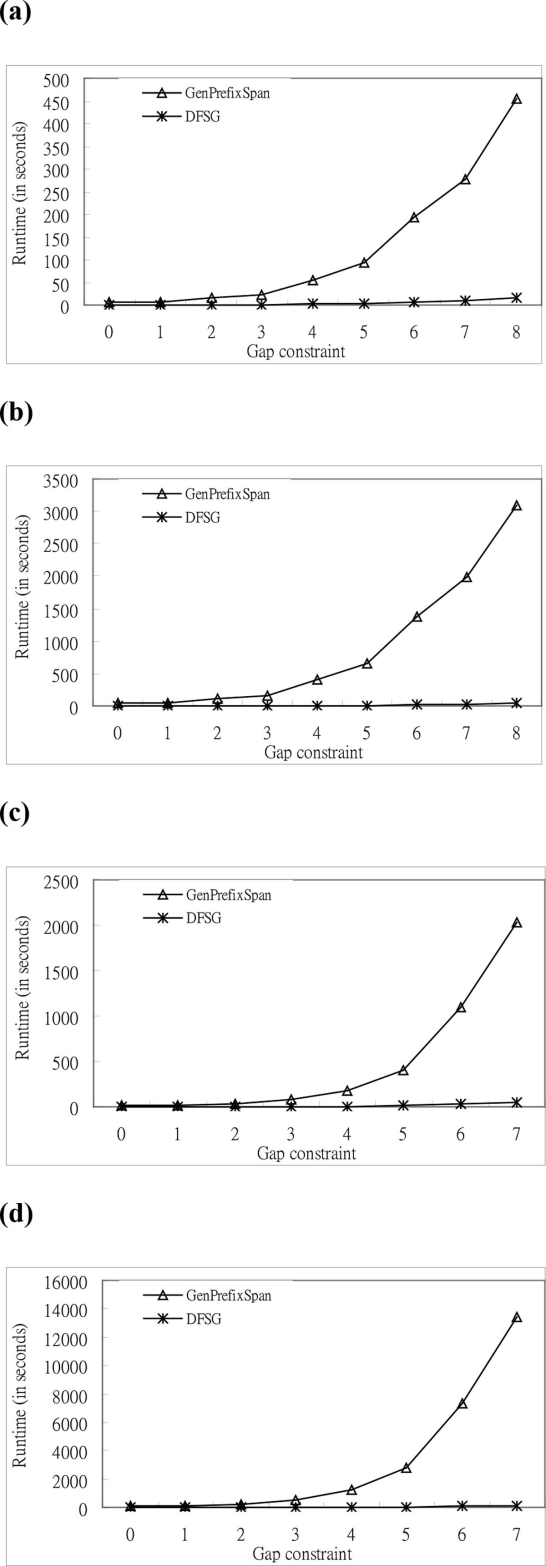
**Comparison of the execution time based on synthetic DNA sequences for the effect of length of gaps**. **a **Execution Time (*L *= 40, *A *= 4, and *N=*1000). **b **Execution Time (*L *= 40, *A *= 4, and *N=*3000). **c **Execution Time (*L *= 50, *A *= 4, and *N=*1000). **d **Execution Time (*L *= 50, *A *= 4, and *N=*3000).

*DFSG *performance and the performance of *GenPrefixSpan *are compared by using gap constraints, number of sequences, length of sequences, and simulated protein sequences. Scalability of the *DFSG *algorithm is also tested. We raise the value of the gap constraint *G*, and the number of letters *A *equals four steadily for simulated DNA data in the experiments of gap constraints. *DFSG *is superior to *GenPrefixSpan *with variable gap constraints according to Figure 6a-d. In the experiments, the lengths of the sequences are forty and fifty; and the numbers of sequences are one thousand and three thousand. The execution time of *DFSG *and that of *GenPrefixSpan *is raised since the probability of finding subsequent frequent items is enhanced, and the number of sequential patterns is increased when we increase the value of the gap constraint *G*.

**Figure 7:**
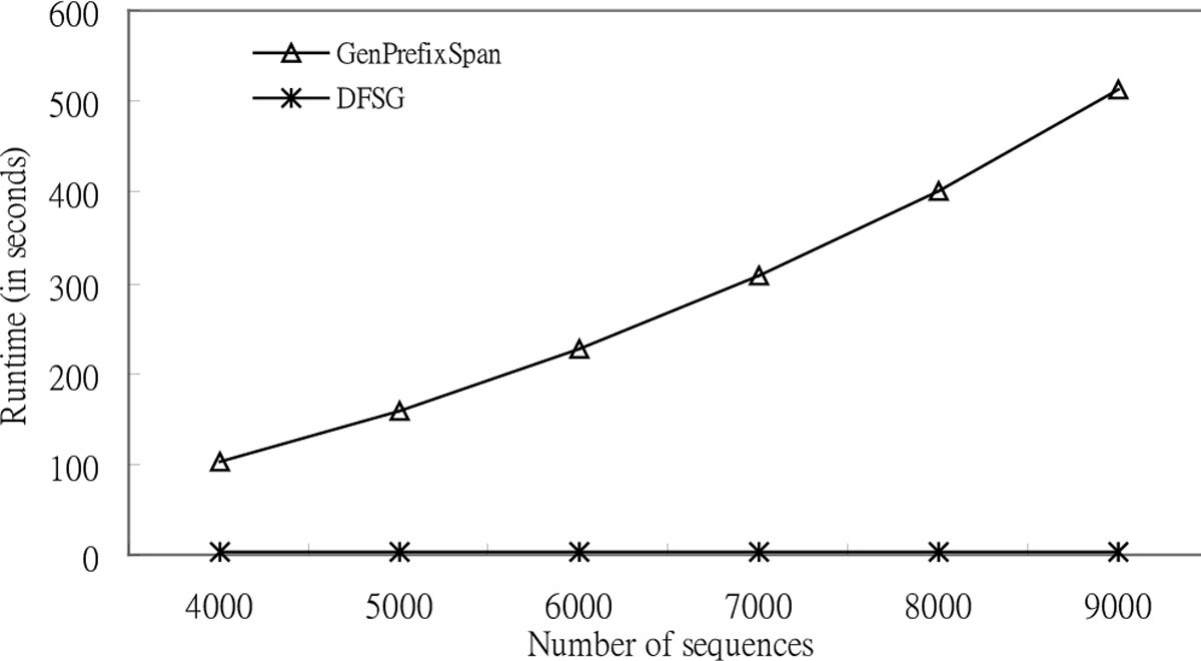
**Comparison of the execution time based on synthetic DNA sequences for the effect of number of sequences**. Execution Time (*L *= 30, *A *= 4, *G *= 3, and *S *= 0.9).

*DFSG *is superior to *GenPrefixSpan *with raised *N *according to Figure 7. In the experiments, the numbers of sequences are four thousand, five thousand, six thousand, seven thousand, eight thousand, and nine thousand; the number of synthetic DNA data is four; the length of the sequences is thirty; the minimum support is zero point nine; and the value of the gap constraint is three. Additionally, *DFSG *is more scalable than *GenPrefixSpan*, although *GenPrefixSpan *is scalable [26]. The runtimes of *DFSG *seem to be a straight line in Figure 7 as a result of the proportional scale. *DFSG *runtimes of Figure 7 are 2.781 s (four thousand sequences), 2.890 s (five thousand sequences), 2.937 s (six thousand sequences), 3.015 s (seven thousand sequences), 3.125 s (eight thousand sequences), and 3.187 s (nine thousand sequences). The variations of *DFSG *runtimes are not obvious in Figure 7. Furthermore, the runtime rates are 37.12, 55.38, 77.24, 102.32, 128.77, and 160.72. The runtime rate rises when the number of sequences gets larger. This experiment confirms that *DFSG *is more efficient than *GenPrefixSpan *when the number of sequences is increased.

**Figure 8:**
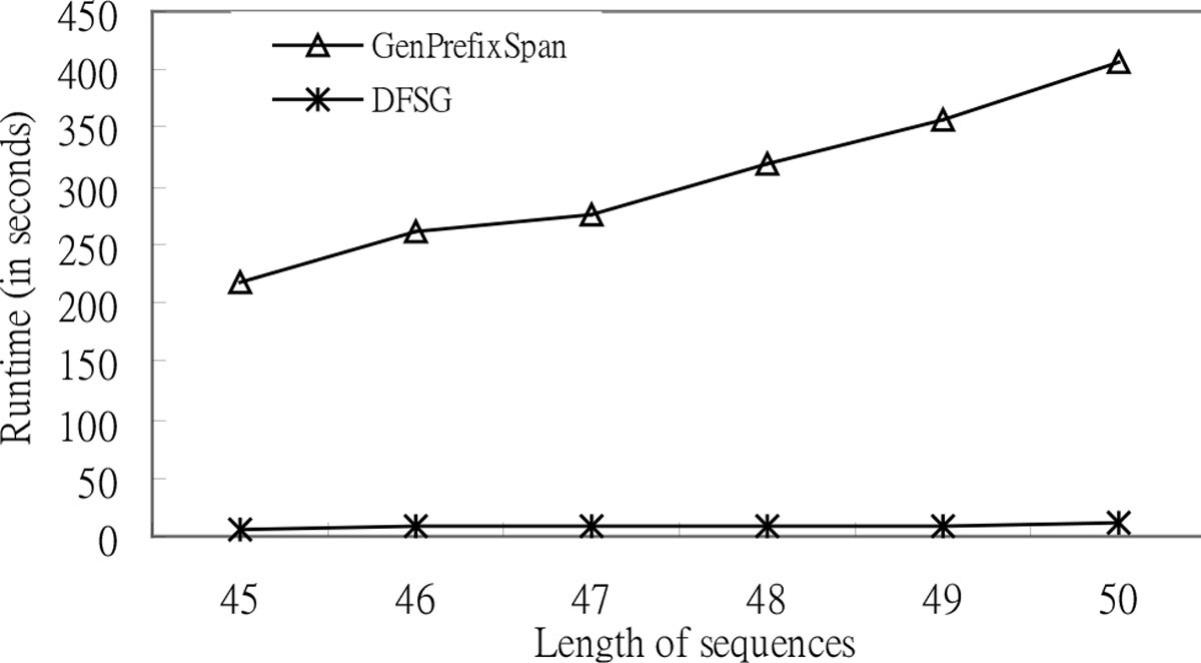
**Comparison of the execution time based on synthetic DNA sequences for the effect of length of sequences**. Execution Time (*A *= 4, *N=*1000, *G *= 5, and *S *= 0.9).

**Figure 9:**
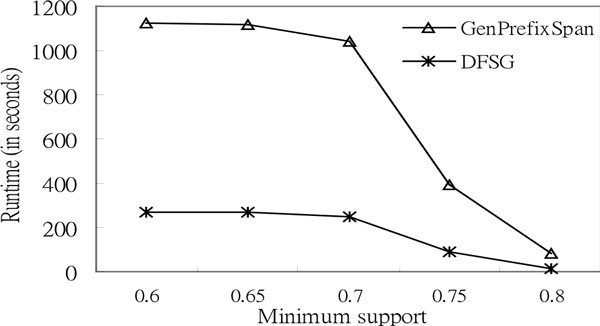
**Comparison of execution time based on simulated protein sequences**. Execution Time (*A *= 20, *L *= 100, *G *= 20, and *N *= 500).

According to Figure 8, *DFSG *outperforms *GenPrefixSpan *with increased *L*. The lengths of the sequences are forty-five, forty-six, forty-seven, forty-eight, forty-nine, and fifty; the number of letters is four; the minimum support is zero point nine; the value of the gap constraint is five; and the number of sequences is one thousand in the experiments. The *DFSG *runtime of Figure 8 rises steadily when the length of the sequences *L *is increased that is compared to *GenPrefixSpan*. As a result of the proportional scale, the runtimes of *DFSG *for this figure seem to be a straight line more if the maximum value of the sequence length is greater than fifty. Additionally, the results of the experiment have already shown that *DFSG *significantly outperforms *GenPrefixSpan *as *L *increases. In the following, we use a different alphabet size to test the execution time of *DFSG *and that of *GenPrefixSpan*. According to Figure 9, *DFSG *mines much faster than *GenPrefixSpan *when the number of letters *A *equals twenty for synthetic protein data. The length of the sequences is one hundred; the number of sequences is five hundred; and the value of the gap constraint is twenty in the experiment of synthetic protein data.

The number of sequences *N *is added from one hundred kilos to five hundred kilos to experiment with *DFSG *scalability. In the experiment, the runtimes of *DFSG *are 36.218, 73.640, 108.390, 152.109, and 206.640 s, and the numbers of sequences are one hundred kilos, two hundred kilos, three hundred kilos, four hundred kilos, and five hundred kilos, respectively. In the experimental results, the execution time of *DFSG *is scalable when the numbers of sequences get larger. The growth rate of *DFSG *runtime is steady. This experiment confirms that *DFSG *has the scalability for large biological data. In the experiment, the number of letters for the synthetic DNA data is four; the minimum support is zero point nine; the value of the gap constraint is three; and the length of the sequences is thirty. The total experiments show that *DFSG *is superior to *GenPrefixSpan *in various features, including synthetic DNA/protein data, real DNA data, length of sequences, number of sequences, and gap constraints.

## Conclusions

Mining sequential patterns of biological sequences is important in computational biology. However, traditional sequential pattern mining methods difficultly cope with biological sequences whose sequence lengths are long, and alphabets are small. Furthermore, gap constraints for motif discovery are also important in computational biology. Therefore, *DFSG *is proposed to efficiently mine motifs of biological sequences with gap constraints. *DFSG *can help biologists discover all-length motifs with gap constraints, and when mining biological sequences, *DFSG *is more efficient than *GenPrefixSpan*. In our future works, we will devise efficient or effective algorithms to help mine biological sequences.

## Competing interests

The authors declare that they have no competing interests.

## Authors' contributions

VCCL carried out this work, conceived of the study, designed the conceptual framework, developed the method, analyzed the results and drafted the manuscript. MSC advised on this work and helped to draft the manuscript. Both authors have read and approved the final manuscript.

## Declarations

The publication costs for this article were funded by the corresponding author.

This article has been published as part of *BMC Systems Biology *Volume 7 Supplement 4, 2013: Selected articles from the IEEE International Conference on Bioinformatics and Biomedicine 2012: Systems Biology. The full contents of the supplement are available online at http://www.biomedcentral.com/bmcsystbiol/supplements/7/S4.
